# CalfSim tool: A free and user-friendly decision support tool for designing and simulating optimized feeding plans for dairy calves—A prediction assessment study

**DOI:** 10.3168/jdsc.2025-0777

**Published:** 2025-07-03

**Authors:** T.E. Da Silva, J.H.C. Costa

**Affiliations:** Department of Animal and Veterinary Sciences, the University of Vermont, Burlington, VT 05405

## Abstract

Optimizing calf feeding strategies is critical for improving performance, health, and weaning transitions of preweaning animals. Despite the updated National Academies of Sciences, Engineering, and Medicine (NASEM, 2021) *Nutrient Requirements of Dairy Cattle*, decision support tools integrating these equations for simulating optimized calf feeding strategies remain limited. To address this gap, we developed and tested the CalfSim, a free, user-friendly decision support tool designed to simulate and optimize feeding plans for dairy calves. CalfSim incorporates inputs that farmers can readily collect, enabling users to establish baseline scenarios, explore “what-if” questions, and evaluate the impacts of nutritional strategies on calf performance and rearing cost. The tool is built based on NASEM (2021) equations, with adaptations for predicting energy-allowable growth and solid feed intake under varying conditions. CalfSim's performance predictions were assessed using data from 27 studies, 24 from the *Journal of Dairy Science* and 3 other unpublished studies (n = 76 treatments, 258 BW observations, and 1,585 calves), demonstrating accurate and precise predictions (root mean square error = 8.56 kg, R^2^ = 0.91, and concordance correlation coefficient = 0.93). However, farm-specific factors (e.g., disease incidence, environmental stress, genetics) can influence predictions, and these factors are not accounted for. This highlights the need for on-farm data to assist users in making more informed decisions (e.g., by comparing scenarios, calculating cost per kilogram of gain). CalfSim provides actionable insights for designing cost-effective feeding plans, making it a valuable resource for dairy nutritionists, farmers, students, and consultants.

## Highlights

• The CalfSim tool offers an easy and user-friendly interface for designing optimized milk plans for dairy calves.
• CalfSim's body weight predictions demonstrate both accuracy and precision.
• CalfSim's predictions should be complemented with on-farm data for optimal application.

## Graphical Abstract

**Figure:**
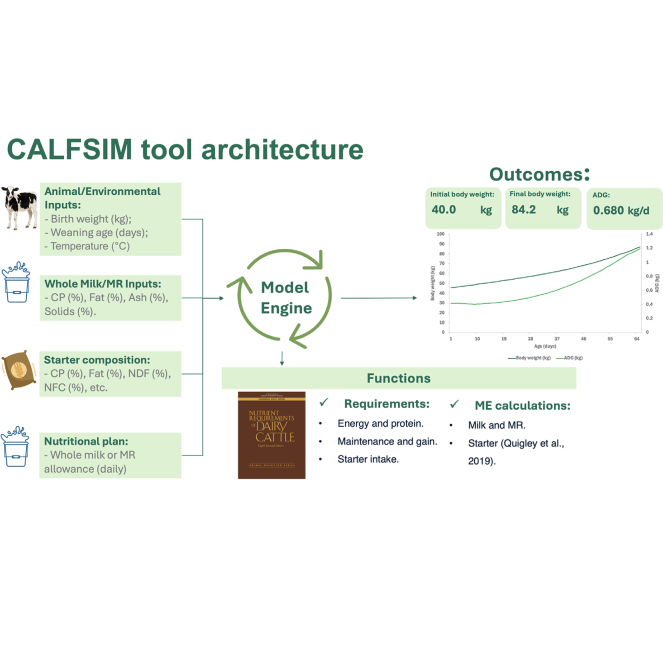
**Summary:** Optimizing milk feeding strategies is essential for calf performance, health, and a smooth weaning. Although the 2021 National Academies of Sciences, Engineering, and Medicine (NASEM) guidelines updated nutritional requirements, few tools integrate these into practical decision support systems. We developed CalfSim, a free, user-friendly tool based on NASEM equations and adapted with other published models to simulate body weight gain, nutrient availability and uptake, and solid feed intake with the objective of being used to optimize calf milk feeding strategies. Built in R using the Shiny app web framework, it allows users to evaluate scenarios using farm-relevant inputs. Here, we validated the model with data from 27 studies and found that CalfSim offers accurate and precise predictions (root mean square error = 8.56 kg, coefficient of determination = 0.91, and concordance correlation coefficient = 0.93), and can be used to support informed decisions for tailored calf nutrition plans. MR = milk replacer.

Optimizing on-farm calf milk feeding strategies is essential, not only for better performance (e.g., weight gain), welfare levels, and health, but also for promoting higher calf starter intake, enabling earlier and smoother weaning transitions (Costa et al., 2019).

The 2021 release of the National Academies of Sciences, Engineering, and Medicine (**NASEM**) *Nutrient Requirements of Dairy Cattle* revised the equations for solid feed intake and the nutritional requirements of dairy calves. Although other desktop applications exist that use nutritional models for dairy calf performance, to our knowledge, there are still very few publicly accessible, web-based decision support tools that embed those simulation equations for optimized calf milk feeding strategies within an intuitive graphical user interface. The development of such decision support tools holds potential to assist dairy farmers and consultants in applying computer-based modeling and simulation techniques—commonly referred to as in silico experimentation—for practical and data-informed farm management decisions (Tedeschi and Fox, 2020). This approach allows for virtual testing of different scenarios, management strategies, or nutritional plans, minimizing risk and enhancing decision-making before real-world implementation.

Recognizing this gap, we developed CalfSim, a user-friendly, intuitive, well-documented, and easily accessible web decision support tool designed to simulate and optimize feeding plans for growing dairy calves during the preweaning and weaning phases. By incorporating inputs that dairy farmers can readily and accurately collect, CalfSim enables users to effortlessly establish baseline scenarios and explore “what-if” questions and scenarios. The tool outputs the effects of various nutritional strategies on calf performance, predicts solid feed intake, calculates important nutritional variables (e.g., daily and total intakes of nutrients, daily total requirements) and key performance indicators (**KPI**), and calculates total cost and cost per weight gain, providing actionable insights for optimized decision-making.

Thus, the objectives of this article are 2-fold: (1) to describe CalfSim, a user-friendly, free-access tool designed to simulate optimized feeding plans for dairy calves, by detailing its fundamental principles and functionalities, and (2) to evaluate its performance through model assessment using input and output data (e.g., calf performance) sourced from the recent scientific literature, with the tool's predictions tested against a dataset comprising 27 independent studies and 1,585 calves.

We developed the CalfSim tool using the package and framework for web application development called Shiny (shiny.rstudio.com; Wickham, 2021) as a backbone, available in the R programming language (version 4.3.3, R Core Team). In addition to the Shiny framework, we also employed the *Golem* package, another R package and framework for building robust production-grade Shiny applications, contained inside a R package (https://golemverse.org/; Fay et al., 2021). For the development of the graphical user interface, the bootstrap 5 framework was leveraged through the *bslib* package. The generation of simulation reports involved the utilization of the *Rmarkdown* package (https://rmarkdown.rstudio.com/), which produced HTML files encompassing all model inputs and outputs.

The development of the CalfSim tool was based on the nutritional requirement equations for maintenance and growth, for both energy and protein, as outlined in Chapter 10 of NASEM (2021). Maintenance requirements (NEM) were calculated as follows: NEM (kcal/kg) = 76.9 × EBW^0.75^ (for preweaning calves), and NEM (kcal/kg) = 97.0 × EBW^0.75^ (for weaned calves), with empty body weight (**EBW**) given in kilograms. Empty body weight (kg) was calculated as 0.91 × BW for calves receiving milk or milk replacer plus solid feed, and as 0.85 × BW for fully weaned calves. Metabolizable energy requirements were calculated by dividing the NEM by the efficiency of ME utilization (**k_m_**). In preweaning calves, k_m_ was assumed constant at 0.69, such that MEm (kcal) = NEM/0.69, where MEm is metabolizable energy for maintenance. For calves fully transitioned to solid feed after weaning, k_m_ was not fixed, but instead estimated as a function of diet energy density, as follows: k_m_ = [(1.1104 × ME) − (0.0946 × ME^2^) + (0.0065 × ME^3^) − 0.7783]/ME.

For energy requirements for gain, an adaptation was made to rearrange the original energy retention equation [i.e., retained energy (Mcal/d) = (EBW gain^1.1^ × (EBW^0.205^)] to EBW gain based on net energy availability and EBW. Specifically, EBW gain was calculated as NE/[(EBW^0.205^)^1/1.1^], where NE represents total net energy available (Mcal/d). This adjustment was made to align the tool with its purpose of predicting daily performance primarily based on nutrient input, particularly energy (i.e., energy-allowable growth).

Total DMI in the tool is calculated as the sum of DMI from the liquid diet and solid feed. All liquid diet offerings were assumed to be fully consumed, but intake was capped at a biologically relevant maximum of 14 L per day—a threshold derived from published studies demonstrating that calves under ad libitum liquid feeding regimens never exceeded this volume (Jasper and Weary, 2002). The DMI from the solid feed (i.e., starter) is estimated using the equations proposed for initial starter intake under temperate and semitropical conditions, both of which were incorporated into the tool. The equations for solid intake (**SI**) were as follows: (1) for temperate conditions (≤35°C), SI (g/d) = −652.525 + (BW × 14.734) + (MEiLD × 18.896) + (FPstarter × 73.303) + (FPstarter^2^ × 13.496) − (29.614 × FPstarter × MEiLD); and (2) for semitropical conditions (>35°C), SI (g/d) = 600.053 × {1 + 14,863.651 × [exp(−1.553 × FPstarter)]} −1 + (9.951 × BW) − (130.434 × MEiLD), where MEiLD = ME intake from the liquid diet (Mcal) and FPstarter = time relative to first offer of starter (d). The tool automatically applies the semitropical equation when the ambient temperature declared by the user exceeds 35°C.

Because daily weight gain predictions are daily, an adaptation in relation to the calculation of ME from solid feed intake was necessary. The gastrointestinal tract of calves is not fully developed during the first weeks of life, and the reticulorumen has limited fermentative capacity (Quigley et al., 2019a); thus, assuming calves could extract the full energy from solid feed in the initial weeks of life would be biologically implausible and inaccurate. To address this, we applied the linear equations proposed by Quigley et al. (2019a), which adjust the digestibility values according to the accumulated intake of NFC, to estimate the nutrient digestibility of the solid feed components. In these equations, digestible crude protein (dCP) is calculated as dCP = 0.707 + 0.0268 × lnccsNFCI – 0.329 × PEL − 0.042 × TEX + 0.107 × PEL × lnccsNFCI – 0.013 × TEX × lnccsNFCI); digestible fat (dFat) is calculated as dFat = 0.883 − 0.021 × lnccsNFCI −0.212 × PEL − 0.380 × TEX + 0.091 × PEL × lnccsNFCI + 0.122 × TEX × lnccsNFCI); digestible neutral detergent fiber (dNDF) is calculated as dNDF = 0.390 + 0.026 × lnccsNFCI − 0.090 × PEL − 0.263 × TEX + 0.078 × PEL × lnccsNFCI + 0.082 × TEX × lnccsNFCI); and digestible NFC (dNFC) is calculated as dNFC = 0.935 + 0.004 × lnccsNFCI − 0.348 × PEL − 0.322 × TEX + 0.134 × PEL × lnccsNFCI + 0.127 × TEX × lnccsNFCI); where lnccsNFCI = natural logarithm of cumulative calf starter NFC intake (kg), PEL and TEX are the form of starter (pelleted and texturized, respectively), and coefficients for TMR = 0. According to these equations, each component's digestibility reaches its maximum only after cumulative NFC intake exceeds 15 kg, indicating that the calves' gastrointestinal tract has matured sufficiently to extract energy efficiently from the starter feed (Quigley et al., 2019b). After estimating the digestibility of the calf starter components and the total digestibility, the ME is calculated based on the NRC (2001) equations (Quigley et al., 2019b).

All equations used in this model were converted into R functions and thoroughly documented, including descriptions of all required argument parameters. This approach ensures easier maintenance and allows for potential future feature additions or modifications. Furthermore, the application was modularized into distinct components to streamline both maintenance and development processes (Wickham, 2021).

The CalfSim tool can be accessed via the Costa Laboratory at the University of Vermont website (go.uvm.edu/calfsim) through the CALFSIM tab. To democratize the use of the tool and increase its uptake, CalfSim is available in English, Spanish, and Portuguese. The text was written in English and translated to the other languages; language accuracy was achieve by requesting alpha testers that are experts in the field and fluent in the selected language to review the text, suggest edits and comments, and approve the final version. Immediately after opening the tool, the user can click on the top right corner of the screen to access and select language options (Figure 1). Once a language is selected, all inputs, results, and reports will be displayed in the chosen language. In the case of the English language, in addition to the international metric system, if the user prefers, there is also the option of the imperial system to view and interpret the outcomes, as well as obtain a report with this system of measurement (top left corner of the Dashboard tab).

**Figure 1:**
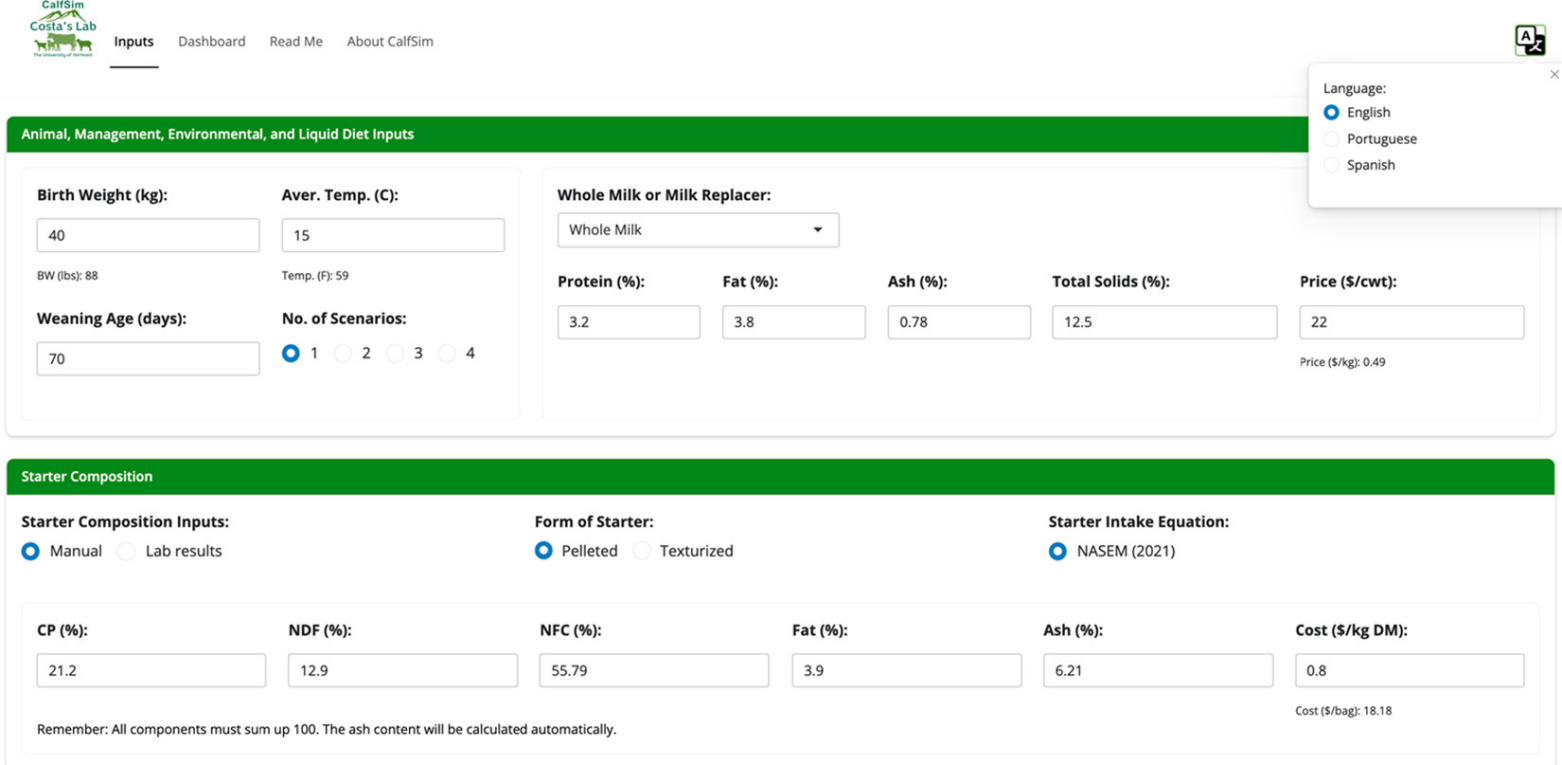
Home page of the CalfSim tool (Inputs tab; found at go.uvm.edu/calfsim), a decision support tool for designing and simulating optimized feeding plans for dairy calves based on NASEM (2021). Default inputs are pre-filled to allow users to use them as reference values.

The CalfSim tool is arranged into 4 navigation tabs: Inputs, Dashboard, Read Me, and About CalfSim (Figure 1). The Inputs tab allows users to enter all the necessary data to run simulations. It is divided into 3 sections:

(1) Animal, Management, Environmental, and Liquid Diet Inputs: Includes the initial weight of the calf (kg; acceptable range: 20 to 60 kg), average temperature (°C; acceptable range: −30 to 50°C), weaning age (days; acceptable range: 10 to 120 d), number of milk plan scenarios to be simulated (maximum of 4), type of liquid diet (i.e., whole milk or milk replacer), along with its chemical composition (all with acceptable range based on expected nutritional levels) and cost.
(2) Starter Composition: Includes the chemical composition of the starter feed (e.g., percentages of DM, CP, NDF) and its cost.
(3) Scenarios for Milk Allowance Plans: Specifies the daily liquid diet allowance until weaning. A preexisting milk allowance scenario based on the developers' milk feeding strategies recommendation is available for the user to test the tool. To run the simulations, the user must click the “Simulate” button and then they will be redirected to the Dashboard tab with the main outcomes.

The tool outputs the impacts of the milk feeding strategies scenarios from the input on calf performance, predicts solid feed intake, calculates important nutritional variables (e.g., daily and total intakes of nutrients, daily total requirements and gain energy and crude protein) and KPIs, and calculates total cost and cost per weight gain. The Dashboard tab displays simulation results and is divided into 3 sections where scenarios can be compared side by side:

(1) KPIs Until Weaning: Presents KPI such as BW (kg), average weight gain (kg/d), the age at which the calf reaches an accumulated NFC consumption of 15 kg, and the feed cost of daily gain ($/kg).
(2) Select Variables to Plot: A plotting area enables users to visualize key variables generated from the simulations, such as BW (kg), weight gain (kg), and starter intake (kg) over the calf's life up to 100 d.
(3) Performance Until Weaning: Provides a table summarizing performance, intake, feed efficiency, and costs for the simulated scenarios. Within the Dashboard tab, the user can download a report (Download button) with comparisons of the main metrics obtained with the simulation of the side-by-side scenarios (e.g., final BW [kg], ADG [kg], feeding cost [$], and cost per kg of gain [$/kg]) and the main performance graphs (e.g., BW [kg], starter intake [kg], and ME intake [Mcal] over time).

Additionally, we created 2 tabs (Read Me and About CalfSim) to facilitate the use and understanding of the tool. The Read Me tab includes both technical details and usage details of the tool to assist in the appropriate use and in the adequate interpretation of the outcomes. It is highly recommended as the starting point for new users. The About CalfSim tab offers additional information about the tool, its developers, and contact details for questions or feedback (feedback forms that are monitored by the authors).

It is important to highlight that the CalfSim tool was developed following the principles of accessibility, easiness of use, interpretation, and practical application, which are identified in the literature as key characteristics for greater adoption and uptake of software-based decision support tools in agriculture (Rose et al., 2016; Baldin et al., 2021). This is demonstrated by the tool's low user effort requirements (i.e., no software installation needed), user-friendly interface (Figure 1, Figure 2), inputs that are easily collected at the farm level, outputs that are straightforward to interpret, and concise documentation to assist users (e.g., the Read Me tab).

**Figure 2:**
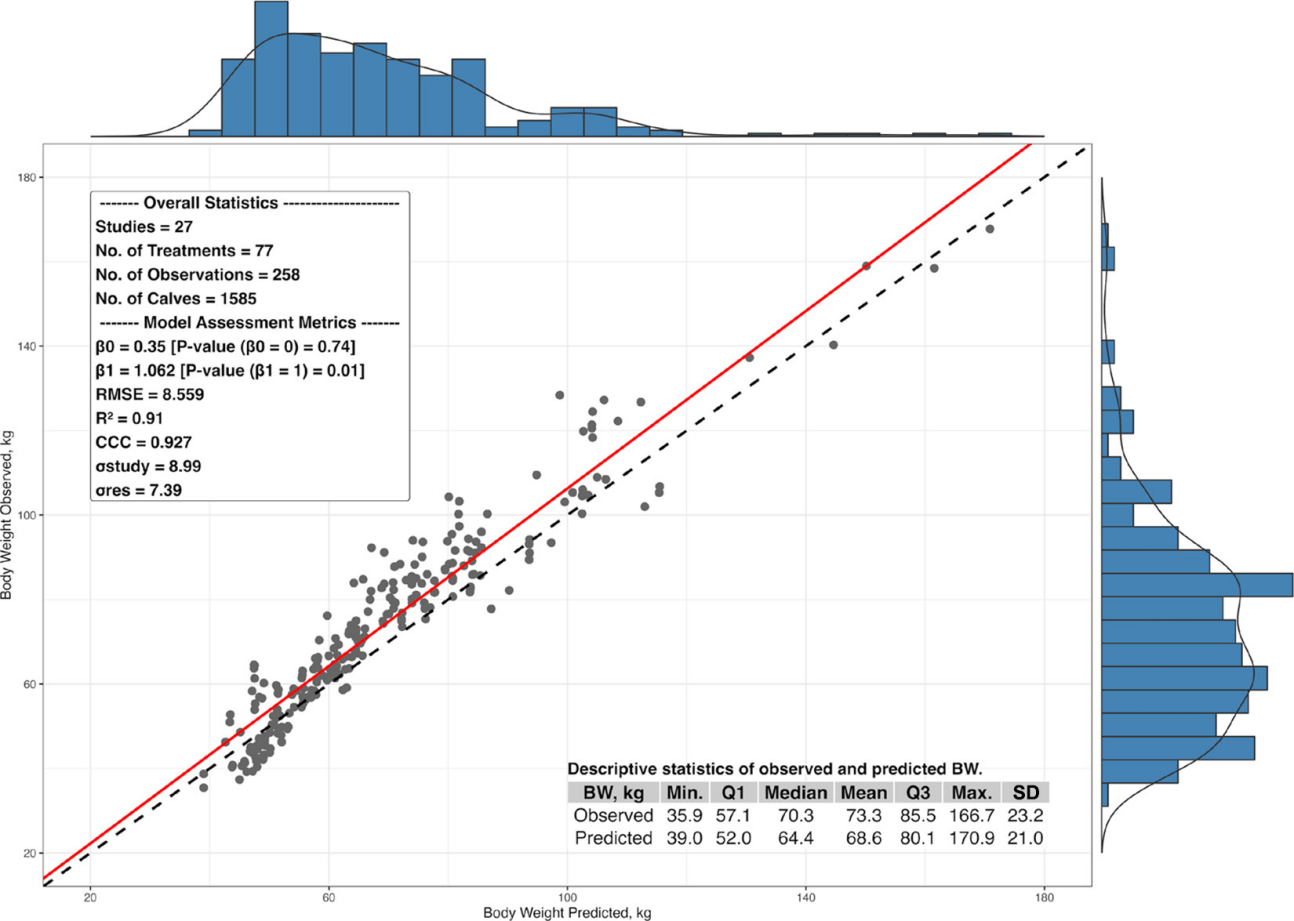
Relationship between predicted BW (kg; x-axis) from the CalfSim tool and observed BW (kg; y-axis) from 27 dairy calf nutrition studies extracted during the literature review. The dashed black line represents the line of equality (X = Y), and the solid red line represents the fitted regression line based on Equation 1. Main model assessment statistics are displayed in the inset box within the scatter plot (upper left). The descriptive statistics of observed and predicted BW, including the minimum (Min.), first quartile (Q1), median, mean, third quartile (Q3), maximum (Max.), and SD, are shown in the bottom right. The histograms above and to the right of the graph show the distribution of predicted and observed values, respectively. RMSE = root mean square error; CCC = concordance correlation coefficient; σstudy = study-level standard deviation; σres = residual standard deviation.

An important part of a decision support tool is its accuracy in predicting scenarios. Thus, to evaluate the performance predictions (i.e., BW) generated by CalfSim, we identified and extracted data from calf articles published in the *Journal of Dairy Science* (**JDS**) from January 2020 to January 2025. We used the JDS advanced search tool and the following terms: (“whole milk” OR “milk replacer”) AND (“dairy calves” OR “dairy heifers”) AND (“performance” OR “body weight” OR “average daily gain” OR “growth), for article title, abstract, and keywords. The primary condition for using the data contained in the papers was to provide the necessary input data to populate CalfSim as well as corresponding performance data for comparison. To be included in the dataset, studies were required to clearly report the milk allowance and milk feeding strategies, the chemical composition of either whole milk or milk replacer, the chemical composition of the solid feed offered, and BW at different ages (days) for each treatment. Studies presenting data only in graphical form, without accompanying mean values, were not included. An exception was made for ambient temperature, where for studies that did not present this data (i.e., 19 studies), we used an average of 20°C to run the simulations. In addition, water and solid feed must have been provided ad libitum in the studies to be included. The JDS search yielded 46 papers. After reviewing the abstracts, materials and methods, and results, out of the 46 papers found, 22 were excluded due to insufficient input or performance data (i.e., those that presented milk feeding plans only as unlabeled graphics, failed to specify starter or liquid diet composition, or displayed BW data in unlabeled figures). Consequently, 24 papers met the criteria for inclusion in the CalfSim predictions assessment, providing all necessary inputs and performance data to run the tool. In addition to the data from the papers available at JDS, we also included 2 studies conducted at Mapleview Agri Ltd. (Palmerston, ON, Canada; J. V. R. Lovatti, University of Vermont, Burlington, VT; in preparation) and 1 at the University of Vermont Dairy Farm Research Center (South Burlington, VT; E. Lopez-Bondarchuk, University of Vermont, Burlington, VT; in preparation). In total, 27 studies (77 treatments, 258 distinct BW data points, and 1,585 calves) were included in the analysis (see Table 1 for descriptive statistics).

**Table 1:** Descriptive statistics (total number of observations, minimum, first quartile [Q1], median, mean, third quartile [Q3], and maximum) calculated for the main variables extracted from the 27 dairy calf nutrition studies extracted during literature review that make up the dataset used to evaluate the CalfSim tool predictions

Variable	Observations, n	Minimum	Q1	Median (mean)	Q3	Maximum	SD
Treatments^1^	77	1	2	3 (2.82)	3	4	—
Calves^1^, ^2^	1,585	10	12	16.0 (20.9)	23.3	62	13
Initial BW, kg	77	34.1	39.2	41.1 (41.8)	44.0	48.9	3.72
Weaning age, d	77	42	56	56 (58.9)	63	90	9.35
Temperature,^3^ °C	8	1.1	14.5	21.5 (18.8)	22.1	24.0	6.14
Liquid diet ME, Mcal/kg	77	3.67	3.71	5.15 (4.63)	5.40	5.76	0.845
Starter ME, Mcal/kg	77	2.91	3.09	3.21 (3.19)	3.29	3.59	0.145
Milk allowance, L/d	77	1.00	3.59	5.00 (5.25)	6.07	13.50	2.543
BW, kg	275	38.19	56.35	70.30 (73.30)	87.22	162.10	22.524

1 For these variables, the minimum, Q1, median (mean), Q3, maximum, and SD were calculated per study collected.

2 Regarding breed, 92% of studies were carried out with Holstein and the remaining 8% were Holstein crossbreeds.

3 Although not all studies presented the average ambient temperature, we used a value of 20°C for the simulations.

The model assessment followed the approach outlined by Tedeschi (2006) and was conducted using the R programming language with customized functions to calculate the main model assessment metrics. Specifically, we used the *lmer* function from the *lme*4 package to regress observed weights against the weights predicted by CalfSim:

[1] $$BWobs_{i}=N\left(\alpha_{j\left(i\right),k\left(i\right)}+\beta_{1}\times BWpred,\;\sigma^{2}\right),$$

where *BWobs_i_* is the *i*th BW observed value following normal distribution with mean *α_j[i],k[i]_* + *β*_1_ × *BWpred* and common variance *σ*^2^, and the values *j*[*i*] and *k*[*i*] represent the specific treatment and study groups for the *i*th observation. The intercept *α_j_* for treatment group *j* is $\alpha_{j}˜\left(\mu_{\alpha j},\sigma_{\alpha j}^{2}\right)$ for treatment within study *j* = 1, …, *j*, where intercept *α_k_* for study group *k* is $\alpha_{k}˜\left(\mu_{\alpha k},\sigma_{\alpha k}^{2}\right)$ for study *k* = 1, …, *k*.

Treatments were nested within study due to the many treatments included multiple bw observations. Within-study treatment variance component was statistically tested against zero via likelihood ratio test. Although treatments were nested in the study effect in Equation 1, this proved to be nonsignificant (*P* > 0.05); therefore, only the random study effect was maintained in the model as random effect.

We found that on average, BW predictions that account for study effects exhibit both accuracy (i.e., β_0_ = 0, *P* = 0.660; and β_1_ = 1, *P* = 0.097) and precision (i.e., R^2^ = 0.91 and concordance correlation coefficient = 0.93, indicating the tool's good predictive performance (Figure 2). These findings demonstrate that the predictions of BW gain in dairy calves in response to various nutritional milk feeding strategies (i.e., different ME inputs from the liquid diet and solid feed) align biologically with expected growth patterns. In addition to accurately representing the average values, the tool's predictions also effectively capture the variability in the outputted data (i.e., BW). This is evident from the histograms and descriptive statistics of the predicted and observed values, notably the similar SD of predicted and observed BW, as illustrated in Figure 2. Therefore, this indicates that the NASEM (2021) model, especially with the adaptations made to meet the specificities of the CalfSim tool, meets the objective of accurately estimating nutritional requirements and growth of dairy calves.

The metrics obtained incorporate the random effects of study, which significantly influence the regression between predicted and observed BW data points $(\sigma_{\mathrm{study}}^{2}$ = 80.80; *P* < 0.05; intraclass correlation coefficient [**ICC**] = σstudy2σstudy2σstudy2+σresidual2σstudy2+σresidual2 = 0.59). The high observed ICC value indicates that measurements taken within the same study—that is, under more similar management and environmental conditions—are more similar to each other compared with measurements taken across different studies. Thus, the significant influence of study effects on CalfSim's BW predictions has important on-farm implications, as the variance component attributable to “study” effectively serves as a proxy for farm-specific variability in predictions. In other words, interstudy differences—quantified by this variance term—give a sense of how much calf performance can vary across farms, even though experimental trials tend to operate under more controlled conditions and may therefore underestimate real-world heterogeneity. Farm-specific effects can be understood as the set of factors inherent to the farm that affect the predictions of BW gain and that are not contemplated in the NASEM (2021) equations and, consequently, in the CalfSim tool.

Disease incidence, such as diarrhea and bovine respiratory disease, is a major factor negatively affecting early life performance in dairy calves (Schinwald et al., 2022). Environmental conditions, including cold and heat stress, also can affect calf performance. Calves exposed to cold stress without adequate heating strategies exhibit reduced weight gain, whereas those in high-temperature conditions may similarly experience growth limitations (Hyde et al., 2022; West, 2003). Management practices such as housing calves individually or in groups further influence performance, with evidence suggesting greater weight gain in group-housed calves (Costa et al., 2016; Donadio et al., 2025). Genetics also play a critical role, affecting maintenance requirements and feed efficiency (VandeHaar et al., 2016). Additionally, calf starter intake is a key driver of growth, influenced by the factors mentioned previously and others such as starter availability, nutritional composition (e.g., protein, fat, and fiber levels), physical form (e.g., texturized or pelleted), and palatability (Quigley et al., 2019a; NASEM, 2021). These factors are not directly accounted for in the CalfSim tool, except for ambient temperature, which influences starter intake prediction and the basal maintenance requirements as per NASEM (2021).

These deviations are somewhat expected, as the tool's foundation (i.e., NASEM 2021) was developed for average conditions (i.e., without considering specific factors such as sanitary management, housing conditions, and ingredient or liquid diet quality). However, from a practical perspective, incorporating farm-specific data alongside the tool's predictions is essential for informed decision-making. Studies evaluating NASEM (2021) equations for milk protein (Binggeli et al., 2024; Carneiro et al., 2025) and fat yields (Binggeli et al., 2023) have shown that farm-level prediction deviations (i.e., underestimation or overestimation) can occur due to factors such as production levels, genetic merit, nutrient utilization efficiency, feed sources, and other farm-specific conditions. Therefore, users should integrate on-farm data to base decisions, especially in cases where deviations are more pronounced. For example, if in a hypothetical user-defined condition CalfSim tool predicts a gain of 0.8 kg/d but the actual observed on-farm gain is 0.6 kg/d, the resulting 0.2 kg/d shortfall should prompt investigations into calf health, housing conditions, and feed quality.

In addition to practical application at the farm level, tools like CalfSim can also be used as effective educational resources for demonstrating nutritional relationship and time series of event to teach students and other professional involved in animal science, agriculture, and related disciplines. By providing outputs such as maintenance and gain requirements, feed efficiency, and other performance metrics, CalfSim helps to illustrate key nutritional concepts. Furthermore, it supports the teaching of strategic feeding plan development for dairy calves through dynamic, visual demonstrations that enhance understanding and student engagement.

As previously pointed out, the limitations of the CalfSim tool are related to the fact that it still cannot use on-farm data to customize performance predictions and make them more accurate and precise. A relevant improvement for future versions would be the inclusion of observed performance data and disease incidence, enabling the adjustment of the model using farm-specific hyperparameters. For instance, such hyperparameters could include adjustment factors for predicted starter intake based on historical feed intake data or growth dampening coefficients related to disease events.

In conclusion, CalfSim addresses a critical need for accessible, science-based nutritional decision support tools for dairy calf nutrition. The tool enables users to simulate and optimize feeding strategies using easily accessible data. Body weight gain predictions obtained with CalfSim were found to be accurate in comparison to published literature, highlighting its reliability and precision, whereas external farm-specific factors, which are not currently integrated into the model, may influence outcomes. As such, being a free-access tool, it represents a practical resource to help design cost-effective feeding plans aimed at improving calf growth, health, and overall farm efficiency.
